# Necropolitical Governance and State-Corporate Harms: COVID-19 and the U.S. Pork Packing Industry

**DOI:** 10.1177/2631309X211011037

**Published:** 2022-06

**Authors:** Ivy Ken, Kenneth Sebastian León

**Affiliations:** 1 Department of Sociology, 8367 George Washington University, Washington, DC, USA; 2 Department of Latino and Caribbean Studies and Criminal Justice Program, 242612 Rutgers University New Brunswick, NJ, USA

**Keywords:** state-corporate crime, crimes of the powerful, legal violence, food crime, business crime

## Abstract

The coronavirus pandemic has magnified the interdependence of the state and corporations in the pork packing industry. In 2020, when over 67,000 meatpacking and processing workers were infected with the virus, the state allowed and encouraged this industry to coerce a racialized workforce to risk their health and lives to slaughter pigs. While it would seem reasonable to call for more regulation to protect labor in this industry, we find by analyzing the state’s actions in 2020 that its interests are too far aligned with corporations’ interests to expect one to police the other. Our analysis underlines the state as a symbiotic partner of corporations, and places workers’ illnesses and deaths in a necropolitical framework that demands attention to the state’s tacit approval of inhumane working conditions, use of law to keep packing plants open, and attempts to limit the liability of corporations for any deaths or illnesses they have caused.

By the end of 2020, at least 67,018 workers across over 1,200 meatpacking and processing plants in the US were infected with the novel coronavirus (COVID-19) (Douglas, 2020). A 61-year-old man named Jose Andrade-Garcia, who worked at JBS plant in Iowa for 20 years, was one of them (Jett, 2020). He became ill after 277 of his JBS colleagues had already tested positive for the virus and eight had died. The League of United Latin American Citizens, an advocacy group, found that workers in Andrade-Garcia’s Marshalltown plant were being made to work “elbow to elbow,” with neither masks, goggles, nor dividers to protect workers. The group filed a complaint on April 1, 2020 with the Iowa Office of Safety and Health Administration (OSHA); the plant safety manager said the complaint “lacked merit.”^1^ Six weeks later, Andrade-Garcia died. His children and grandchildren set up a GoFundMe page to help transport his body for burial in Michoacán, Mexico, where he had planned to return after retirement to care for his elderly mother. Along with Andrade-Garcia, 318 meatpacking and processing workers around the US died due to coronavirus in 2020 (Eller, 2020; Jett, 2020). These deaths and infections were preventable. Is anyone responsible? In complementing contemporary perspectives on state-organized race crime (see Ward, 2015) and legal violence (see Menjívar & Abrego, 2012), we examine whether Andrade-Garcia and his colleagues’ deaths—and the privileges of meatpacking corporations—are “necropolitical” in nature, which is to say, a function of how state-corporate symbiosis and political economic forces shape whose lives are worth protecting and whose lives are disposable in service of capital interests.

High rates of infection and death by COVID-19 among meatpacking workers prompted some plants in the United States to temporarily shut down in 2020, setting off a chain of dilemmas for human, corporate, and animal bodies. For the humans, temporary shut downs were temporarily protective; some of the shut downs lasted one day. For the corporations, shut downs were potential profits lost. The CEO of the National Pork Board, in reflecting on the toll of the pandemic, commented in June, “We can never get those production days back again” (Shike, 2020). For the animals, normally bred, raised, and slaughtered at hyper-efficient speeds and at industrial scales, the utilitarian justification for their inhumane treatment no longer applied. Farmers who raised them were left without industrial buyers, and many had to kill their pigs. Some shot the animals individually. Some pumped carbon dioxide into their feedlots. A handful of plants in Minnesota and Iowa were converted to “euthanasia facilities” where “depopulation methods” included ventilation shutdown (VSD, or death via high temperature and low oxygen). Over 10 million hogs were terminated in this period, their bodies “composted” using wood chippers and mostly dumped in large fields (Corkery & Yaffe-Bellany, 2020; NPPC, 2020). While this has led to a potential $5 billion shortfall for pig farmers, a rise in retail prices justified by the temporary shortage will likely result in increased profits for pork packing companies (Day, 2017; Nepveux, 2020).

In normal times, the swine industry in the United States takes 32.4 billion pounds^2^ of pigs per year—or about 130 million 280-pound animals—and commodifies their flesh and organs as profitable output for both domestic consumption and international export (USDA & Haley, 2019; USDA/NASS, 2020). As inputs, the meat industry also requires approximately 40.4 million pounds^3^ of human flesh—or nearly a quarter of a million people—in the form of frontline laborers.^4^ As an historically unprecedented feat of industrial engineering in its own right, these inputs (i.e., employees) are processed through rationalized domestic and international processing systems, ostensibly designed to feed people. The hyper-efficient breakdown of pig parts is conducted primarily for the hyper-efficient buildup of corporate profits. While a single meatpacking plant can generate over $2 million in revenue per day, and the CEOs of meatpacking companies pocket about $5,000 per hour, the full-time frontline employees of these multinational behemoths average $29,629 per year (about $114 per day) among reliably documented workers (BLS, 2019; Day, 2017; KATV, 2019).

Critiques of the meatpacking industry might be low-hanging fruit, considering the varied ways it has been scrutinized and beleaguered for well over a century, most notably through the 1906 publication of Upton Sinclair’s *The Jungle*. Industrial meat production has long been associated with a wide range of controversial phenomena, including threats to consumer safety (Richtel, 2019); anti-trust policy and litigation (J. Hawley & T. Baldwin, personal communication, 2020; Kelloway, 2008; Senate Judiciary Committee, 2002); unscrupulous lobbying against environmental and public health protections (Ashwood, 2019; Pew Charitable Trusts, 2008); ecological harm (Davis, 2018; Thu, 2010); and systematized disregard for animal welfare (Negowetti, 2018).

Our focus is not on the well-documented actions in this industry that are legally recognized as criminal or administrative violations, however. Instead, we focus on the legal violence that is systematically perpetuated against workers in this industry—violence that is organized by the state and legitimized via law and policy (Alvord et al., 2018). We integrate labor studies with critical criminology to understand the necropolitical dimensions of state-corporate harms against workers. In particular, we analyze the specific ways the highly profitable US pork industry has actively relied on the state to secure a racialized, immigrant labor force kept precarious through legally sanctioned systems of confinement and coercion. Our novel contribution is an analysis of recent—rather than historical—primary material to determine how the state structured and facilitated legal racial violence^5^ that produced both symbolic and actual disposability of the human beings whose labor generates the industry’s profits.

In what follows we describe the structure of the US pork packing industry and our analytical approach, followed by a theoretical discussion locating the criminogenic relationship between corporations and the state. Our analysis follows, with attention three specific arenas in which active disregard for pork industry workers’ lives during the COVID pandemic demonstrates the necropolitical approach of the state: the planned failure of OSHA to require the industry to protect workers; President Trump’s Executive Order to keep meatpacking plants open during the pandemic; and the attempts of the Senate Majority Leader to limit the liability businesses would face when workers become infected and die.

## Structure of the Pork Packing Industry

The three largest pork processing companies in the US reported $40 billion (Tyson Foods), $14 billion (Smithfield Foods), and $49.7 billion (JBS) in revenue in 2018. These three companies process fully two-thirds of the hogs slaughtered each year (USDA & Haley, 2019). The pork slaughter and processing industry has long been concentrated, but has become even more so since the late 1970s through major mergers and the accretion of individual and regional plants (Pacewicz, 2013; C. Ward, 2010). Smithfield Foods—which became so profitable it recently “sold itself” for nearly $5 billion US in the biggest acquisition of a US company by a Chinese company ever (Manning, 2016)—has been particularly aggressive in vertically integrating the industry. The result has been the acquisition of entire farming operations, the expanded use of confined animal feeding operations (CAFOs or “factory farms”), and the rapidly diminished viability of small hog farmers. Labor costs, which are kept low by locating plants in states hostile to unions and by hiring from a supply of vulnerable, racialized immigrant workers (see Ken & León, n.d.), make consolidation through acquisition highly appealing.

The collateral consequences are significant. Increased consolidation is disastrous for farmers who must operate under conditions set largely by and for the benefit of the corporate meatpackers (J. Hawley & T. Baldwin, personal communication, 2020; Pew Charitable Trusts, 2008; Woodall & Shannon, 2018). This was not always the case. While the slaughter of pigs in the colonial and early republic periods occurred on homesteads, yards, farms, or plantations—where human beings were enslaved but pigs foraged freely in forests (Otto, 1987)—the activity was industrialized in the late 19th century and located in cities such as Chicago, Cincinnati, and Omaha. Communities there generated what sociologists John Brueggemann and Cliff Brown call “a triumphant legacy of multiethnic union organizing,” in which 90% of meatpacking workers were covered by union contracts (2003, p. 334). Decades of strikes and strike breaking, federal deregulation, and a shift to investor-centered profit strategy—along with the combined technological contributions of highways, transportation refrigeration methods, and disassembly line efficiencies—resulted in a tectonic shift away from raising, slaughtering, and packaging pork in major population centers in the 1980s and toward increasingly concentrated rural and suburban packing plants (Artz et al., 2010; Pacewicz, 2013). While the industry is extensively regulated by state and federal agencies, it is food safety—not working conditions, compliance with immigration laws, or the industry’s anticompetitive tendencies—that receives the most regulatory scrutiny (Human Rights Watch, 2019; North Dakota State University, n.d.).^6^

In the US, meatpacking companies have demonstrated an uncanny ability to secure a labor force from “a steady supply of people willing to perform one of the most dangerous jobs in the country, for the lowest wage” (Hiddema, 2013, p. 19). Reliable statistics on the ethnic and racial makeup of the frontline pork packing workforce are elusive, in light of plant- and location-specific variation in employment and record-keeping (e.g., Gabriel, 2006), the extraordinary level of turnover (Fitzgerald, 2010), and the industry’s surreptitious employment of workers without visas. Still, it is clear that most current frontline meatpacking workers are not white. The Center for Economic and Policy Research, using 5-year estimates from 2014 to 2018 from the American Community Survey, finds that 44% of current meatpacking workers in the US are Latinx and 25% are Black. The general consensus is that well over half of frontline workers—and in some plants, more than 90%—are immigrants (Fremstad et al., 2020). Race, ethnicity, and residential status functionally grease the wheels of hierarchical labor stratification—a process on which capital depends. Intra-plant variation among specific jobs tells a chapter of this story. Supervisory jobs are disproportionately held by US-born white men; dirty, grueling jobs go to men of color; and the most ancillary and low-paying jobs going to US-born and migrant Latinas, Asian-American women, and Black women (Horowitz, 1997; LeDuff, 2000). Race, class, and gender produce conditions favorable to a de facto caste society within the occupational structure of specific facilities.

Across the board, companies that employ pork packing and processing workers have deliberately designed jobs to require as little skill as possible, and used race, ethnicity, gender, and immigration status as ways of dividing up tasks, sowing discord among workers, breaking unions, and ensuring a continuous supply of replaceable labor. The companies compete, in the words of one investigative reporter, in “a never-ending global scramble for low-skilled labor” (Grabell, 2017). While vegan and anti-animal cruelty organizations have effectively communicated the inhumane fates of the animals that people eat, relatively less prevalent are depictions of the confinement and structural violence experienced by the workers themselves, who spend their days clipping pigs’ snouts, chiseling their cheeks, scooping out their eyes, and carving out their tongues, all under the imperative of speed and efficiency (Genoways, 2014). It is the specific context of legal structural violence against pork packing workers during the coronavirus pandemic which is our primary focus.

## Case Study Parameters and Interpretive Approach

We analyze the state actions and inactions in 2020 that have allowed the pork packing and processing industry to coerce an already vulnerable racialized workforce to further risk their health and lives in unsafe workplaces during the COVID-19 pandemic. By selecting the case of pork packing, we followed Stake’s (2005) identification of a single case as a system, and undertook to find the coherence and sequence among the actors, processes, institutions, and mechanisms within that system. By identifying pork packing as an instrumental, rather than intrinsic case we sought not to identify what is unique about the industry, but rather, to understand it as an exemplar site of the state-corporate coordination and symbiosis that produces a highly concentrated and exploitative structure in the form of a racialized immigrant labor supply.

We pay particular attention to the US pork processing industry during the (long and ongoing) moment of COVID-19 because it (1) involves the state at the national, state, local, and international levels, (2) has taken on its current dimensions on the basis of a history built on both state interference and state failure to intervene, (3) is a site in which a highly vulnerable, immigrant, racialized labor force generates surplus value for multi-billion-dollar multinational corporations, (4) and has been structured around the confinement of human beings inside meatpacking plants, akin to prisons, detainment centers, and nursing homes. This makes the pork processing industry particular when compared to other workplaces, but also ordinary under a late capitalist economic system and an oligarchic political system in which the dynamics of state-corporate symbiosis can be both described and then explained.

Because our analysis focuses on what transpired for workers in pork packing plants in 2020, we relied on newspaper articles, magazine articles, blogs, non-profit organization reports, industry marketing, US Senate hearings, Presidential executive orders, US Chamber of Commerce communications, union positions, and published survey research results from that year. We sought to consider sources from multiple vantage points, including materials produced by the industry, unions, the state, interest groups, non-profit organizations, and journalists. Our analysis of historical patterns in the pork processing industry for context was based primarily on peer-reviewed journal articles, scholarly and popular books, and the direct text of federal and state laws and regulations. We also assembled current workforce and industry data directly from the US Bureau of Labor Statistics and the US Department of Agriculture.

Our analysis relies on an interpretive approach, which demands more than a report of “findings” or a compilation of identified trends (Schwartz-Shea, 2012). Rather, we employed a theorized attention to the consistencies and contradictions that would allow us to understand how the US state in 2020 contributed to the coercion and confinement of an already precarious work force during the coronavirus pandemic. We focus on the course of infections and deaths that have wracked the industry, the executive order to keep meatpacking plants open, and the attempts to restrict legal liability for the deaths of pork packing workers. Ultimately, our approach is consistent with the logistical and political challenges inherent to studying elite deviance and crimes of the powerful (e.g., corruption, white-collar and state-corporate crime), where the phenomena in question are often systematically omitted from the formal purview of criminological attention and regulatory measurement (Barak, 2017; Geis, 2016; Gottschalk, 2021; Hall & Winlow, 2015; Lasslett, 2018; Tombs and Whyte, 2015; Yeager, 2009; see also León, 2021, p. 39, 51).

## The Interdependence of Corporations and the State

Interdependent interests and incentives connect state affairs with corporate affairs. By “the state,” we refer to a bureaucratic, fragmented, and crowded ensemble of institutions and actors that organize social, economic, and political relations of power, despite competing priorities, sometimes zero-sum resource streams, and wide-ranging forms of intra- and inter-group conflict and compromise (Tombs & Whyte, 2020). In actually existing capitalism, the state ensures the successful accumulation of capital and incentivizes acquiescence to the manner in which that is accomplished (Amable, 2017; Evans, 1995; Streeck, 2018). This does not mean the state acts uniformly or even coherently according to the desires of every capitalist and corporation. Rather, the state prioritizes the interest of “capital in general,” whether that requires containing labor or moderating the actions of particular corporations within a specific industry or subsector (Block, 1977; Jessop, 1990). Empirically identifying specific maneuvers through which the state accomplishes this is what leads us to claim that state-corporate “success” is predicated on the violently cultivated and sustained precarity of the lives of workers

It would be easy to imagine the relationship between the state and corporations as fundamentally antagonistic, given the rehearsal of neoliberal rhetoric demanding that the government “get out of the way” and let corporations do business (Bernat & Whyte, 2017). In practice, this relationship is symbiotic. The state makes corporations possible. It encodes the legal basis on which corporations can function and be given rights, and provides the monetary infrastructure within which corporations can engage in production and exchange (Jessop, 1990). Conversely, corporations allow a state to accomplish the accumulation of capital that finances that state’s power. “In capitalist social orders,” Bernat and Whyte argue (2017, p. 77), “states play a creative and enabling role for regimes of capital accumulation; corporations are the key institutions in realising capital accumulation.” The state-corporate symbiosis in the US depends on legitimacy garnered by laws, agencies, and government actions that ostensibly protect workers from harm, while still securing a labor force that advances capital accumulation projects as efficiently as possible.

“Harm” is the word many critical criminologists use to mark the important distinction between “crimes,” which are definitionally bound by the politics of criminal law creation and thus omit harmful practices that are not legally recognized as criminal (Matthews & Kauzlarich, 2007). As covered extensively in the history of critical criminology (see Chambliss, 1975; Schwendinger & Schwendinger, 1970), concepts like “felon” and “felony” must be socio-legally created before they can be said to exist (Hillyard & Tombs, 2007, p. 10). State actors decide what is criminal and how to enforce codified criminal law, so the injuries and deaths the state commits (e.g., police killings of Black Americans, planned executions, war) are rarely defined or successfully prosecuted as legal crimes (Chambliss et al., 2013).^7^ Terms like “harm” or “legitimized crime” thus refer to the range of activities that are not formally prioritized by actors in power-wielding institutions (see Michalowski, 2016). Deaths and illnesses that the state interprets as accidents or byproducts of capital accumulation—inside workplaces and also as a result of polluted air, water, land, and species—are overwhelmingly handled without burdening corporations for their costs, let alone for the moral debt or criminal liability of each life altered. Even in cases where, in order to bolster legitimacy, the capitalist state does define particular causes of workplace injury and death as criminal, it is unlikely to regulate those harms as such or enforce its own regulations in any comprehensive manner. Our work is consistent and complementary to canonical scholarship on state-corporate harms, including the complicity continuum of Kauzlarich et al. (2003), which articulates how organizational goals generate state-initiated and state-facilitated crimes, including crimes of omission. State-corporate crime is not a hyphenated umbrella term of convenience, but a more accurate articulation of how corporate power is configured and perpetuated. As Tombs and Whyte (2020) have argued, corporate harms occur not because of a “breakdown in the regulatory function of states; they occur as part and parcel of a process of corporate power-mongering, and, in the main are tolerated and encouraged by states.”

It is true that—with great goading, agitation, and collective action from unions—the state has been a source of meaningful improvements to the lives of workers. These include state and federal minimum wage laws, the Occupational Safety and Health Act, social security, the Equal Pay Act, and thousands more laws, court decisions, executive orders, agency establishments, forms of federal and state aid, cases prosecuted, and regulations to mitigate the most obvious of foreseeable harms. Given these real gains, it might seem that the obvious answer to the state’s lack of regulation of workers’ interests is more and better law and regulation. Political economists, though, remind us of the importance of legitimacy in this calculus (Evans, 1995). The state has to both enable capitalist activity and appear legitimate and neutral—or even antagonistic (Michalowski, 1993). Securing legitimacy is an achievement that must be continually and actively pursued and enacted, through such activities as seeming to “do something” about the harmful effects of capital accumulation projects (Dello Buono, 2015; Jessop, 1990). Critical criminologists, such as William Chambliss, point to legal remedies not as real solutions but as piecemeal attempts to maintain legitimacy and avoid confronting the core contradictions of capital and its corresponding political economic dilemmas. State intervention is part of a dialectic process of addressing context-specific conflicts in a way that will “maintain the capitalist system without fomenting a revolution” (Chambliss, 1979, p. 153). Building on Chambliss, Tombs and Whyte (2020) point out that,

The regulatory regime to which corporations are subject is put into place and maintained by the state. To the extent that this regime allows harm (through its very nature) or crime (through its non-enforcement) to be produced, this should lead us to understand such harm or crime as “state-corporate” in character. In this sense, all forms of corporate crime and harm implicate the state to a greater or lesser extent. In this context, should we rely on states to regulate corporations?

If the answer to their question is no, then the consequences of these symbiotic interdependencies must be audited in full. The state—in addition to its “twin tasks of legitimation and accumulation” (Michalowski, 1993, p. 93)—has sovereignty to determine who lives and who dies. The philosopher Achille Mbembé, engaging with the work of Franz Fanon and Michel Foucault, considers the conditions under which death, itself, is administered by the state, in its “capacity to define who matters and who does not, who is *disposable* and who is not” (2003, p. 27, emphasis in original). While his concept of “necropolitics” is built on harrowing examples of war, slavery, genocide, and occupation, the moment of the coronavirus pandemic as it played out in the United States in 2020 gives us reason to consider how the confinement of workers in dangerous workplaces informs “murderous functions of the state” (2003, p. 17). When workers die as a result of harm perpetrated by corporations, the public does not necessarily consider their deaths acts of the state. This is not to suggest that corporations are not responsible for workers’ deaths—quite the contrary. It is, rather, to underline the state as a necessary and symbiotic partner of corporations, and to then place workers’ deaths in a necropolitical framework that demands attention to the needs of the state in “resolving” conflicts over companies’ inhumane working conditions by failing to regulate them, invoking the law to coerce workers to remain in harmful workplaces, and attempting to limit the liability of corporations for any deaths or illnesses they have caused (Harlow, 2011; Michalowski, 2020).

Sociologist Jin-kyung Lee (2010, p. 6) describes “necropolitical labor” as “a job that can be carried out only by necessarily risking one’s life.” She identifies military labor as an obvious example of this, emphasizing how soldiers directly carry out the will of the state. Necropolitical labor is also found in the manufacturing sector, such as in meatpacking plants where threats of acute and long-term injury and death are commonplace and the state regularly looks the other way. In this paper, by demonstrating the symbiosis of the state with pork packing corporations, we hope to make the will of the state—as carried out by a largely non-unionized, racialized workforce in a highly concentrated industry—apparent.

The moment of COVID-19 provides a telling indictment of necropolitical state-corporate symbiosis in the pork packing industry. We identify the (in)actions of state and federal offices of the Office of Safety and Health Administration (OSHA) in pork packing plants during the pandemic, an executive order^8^ that kept pork packing plants open, and the attempts of state and federal legislators to limit legal corporate liability for pork packing workers’ illnesses and deaths. In this we seek to understand how state-corporate symbiosis in the US pork industry bolsters capital accumulation using forms of social control to determine who can remain healthy and who is disposable.

## Confinement and Coercion During Covid-19

Throughout 2020—and for 15 days in April in particular—the state intervened in the pork packing industry in ways that are simultaneously unprecedented and completely consistent with previous actions. Here we explore federal agency guidelines for worker safety, barefaced public cooperation between meatpacking companies and the executive branch, and legislation passed in the biggest pork packing states to limit the liability of meatpacking companies for the deaths and illnesses of their workers.

### Infections Seize Pork Packing Workers: OSHA as a Failed Safety Net

The first cases of COVID-19 infection in the United States were formally detected in February 2020, shortly after the World Health Organization had declared a global health emergency. By March 9, the US government had issued guidelines on social distancing and personal protective equipment. Less than two weeks later most states in the US issued stay-at-home directives, and almost immediately 10 million people in the US were out of work. Pork processing plants continued production.

Managers suspected there were infections within their meatpacking plants as early as mid-March, when they started asking workers if they had experienced fever or coughing (UFCW, 2020; Valdivia & Margolies, 2020). Still, pork packing companies did not begin scaling back slaughter until early April, and fully closing plants starting April 7 (Meatpoultry, 2020). Cases of infection among workers in these plants were causing “spikes” in the states where they are located: 277 cases in Iowa (across only two plants); 239 in Minnesota; 669 in Nebraska. In Sioux Falls, South Dakota, the governor asked the head of the Smithfield plant there to stop production after more than 800 workers tested positive.

The Centers for Disease Control and Prevention (CDC) clearly stated in April that “Congregate work…locations are at increased risk for infectious disease transmission including respiratory illness outbreaks,” and that meatpacking facilities were of particular concern (Dyal, 2020). Between April 9th and 27th, COVID-19 was diagnosed in 4,913 workers among 115 facilities across 19 states. The CDC specifically remarked on the harms of the reward structures at these facilities: “Among workers, socioeconomic challenges might contribute to working while feeling ill, particularly if there are management practices such as bonuses that incentivize attendance.” Far from incentives, many workers from crowded plants who reported that their employers had failed to provide masks said they were simply told to keep working (Telford, 2020a). “If you’re not in a casket, they want you there,” said one former Smithfield worker interviewed in the *Washington Post*. In the plants the CDC personnel visited, they noted that “the pace and physical demands of processing work made adherence to face covering recommendations difficult.” By April 27, the CDC had recorded 20 confirmed coronavirus deaths among meatpacking employees.

OSHA took action on April 26. Together with the CDC they issued interim guidance for meat and poultry processing facilities (OSHA April 26, 2020). Their recommendations included providing protective equipment, hanging informational signs about sneezing etiquette, removing fans, and separating workers by curtains. OSHA also recommended that meatpacking companies encourage workers to walk in single-file lines, monitor each other on the floor, and travel to work without carpooling (CDC, 2020).

These tepid recommendations largely focus on the behavior and agency of employees and not on the built environment of the workplace. By releasing guidance rather than minimum standards or mandatory regulations, OSHA failed to intervene in a meaningful way to protect workers. Companies have no legal obligation to structure their workplaces to maximize workers’ safety on the basis of these recommendations, or any regulatory incentive to shut down production indefinitely to keep employees healthy (and alive). In support of this approach the US Chamber of Commerce argued in a letter to federal, state, local, and county leaders that US companies were “concerned by the push by some to impose new regulatory requirements rather than rely on guidance and best practices.” If federal and state governments took a “regulatory approach,” the Chamber said, companies would have to endure “government bureaucrats” issuing fines in the apparently absurd cases “when they find a sneeze guard out of place, an employee using the wrong mask, or two employees 5 feet 10 inches apart, not the mandated six feet” (U.S. Chamber of Commerce, personal communication, 2020). The coronavirus response was trivialized by framing otherwise commonsensical mitigation efforts as “overreach” by the “nanny state” (Calman, 2009; Pettit, 2015).

One former OSHA official critiqued the agency, arguing that the pandemic is “the most massive workers’ safety crisis in many decades, and OSHA is in the closet. OSHA is hiding” (CBS News, 2020). Further, OSHA reportedly reacted very slowly to respond to workers’ concerns. In one Tyson Foods pork plant, for instance, where workers were worried for their safety,

it took the Iowa division of OSHA nine days to seek a response from Tyson and eight more to get a reply. The state [OSHA] agency ultimately found Tyson’s voluntary efforts to improve social distancing at the Perry plant were “satisfactory” and closed the case without an inspection. A week later, 730 workers—almost 60% of the workforce—had tested positive. (CBS News, 2020)

At the federal level, what appears to be incompetency could be the result of understaffing. In 2020 OSHA employed the lowest number of inspectors since 1975. Leadership roles also remained unfilled for the duration of Trump’s presidential administration. The position of Assistant Secretary of Labor for Occupational Safety and Health, for example (the director of the agency), had no permanent appointee during Trump’s term. Companies, then, were asked to police themselves: “Agency understaffing led to OSHA issuing an April 13 announcement that it would try to deal with coronavirus complaints informally through employers conducting their own investigations, rather than OSHA sending its investigators” (Boullt, 2020).

One former OSHA official with expertise on meat processing plants remarked that, “These outbreaks that have sickened thousands and killed dozens were not inevitable in the meat industry” (Telford, 2020b). However, the typical response of OSHA at the state and federal levels—in times of pandemic and otherwise—is to “work with” employers, and if OSHA inspectors are presented with evidence that companies are “doing their best” to eliminate hazards, the agency will typically not issue citations. Often, it is only after a worker is injured—or dies—that OSHA will conduct an inspection and potentially issue a citation. In these cases, companies usually agree to fix the problem and face no significant penalty.

Trying to bring the state onto their side, workers at one Smithfield plant in Missouri filed suit against the company (Doe, 2020). They did not seek monetary compensation for harms. Rather, the workers plainly asked that the company provide the equipment and infrastructure that OSHA recommended. A judge dismissed the case, arguing that OSHA was in a better place to ensure compliance and that plaintiffs had not demonstrated “irreparable harm” (Margolies, 2020). In assessing the case, the judge said, “the Court must determine whether Plaintiffs will suffer an actual, imminent harm if the injunction is denied. This is not the same as analyzing whether employees risk exposure if they continue to work.” On the day workers filed their suit, there were 1,950 documented cases of COVID-19 infection in meatpacking plants in the US. Within two weeks of the judge’s decision, there were over 16,400 cases of infection—“a more than eight-fold increase in less than a month.” By that time, 66 workers had died of the infection (Food and Environment Reporting Network, 2020).

### Executive Order as State Violence

Lax oversight of severely confined workers during a pandemic is one thing; an Executive Order to coerce workers to continue working, echoing the sentiments of a major meat industry executive, is another. Consistent with the research of Alvord et al. (2018), we analyze Donald Trump’s April 28, 2020 Executive Order as facilitating legal violence.

On April 27, John Tyson, the chairman of Tyson Foods’ executive board, paid to publish a full-page ad in two national newspapers. By this time, 16 (out of more than 900) meatpacking plants had temporarily closed. “The food supply chain is breaking,” Tyson’s plea said. Pork production and slaughter was actually only down 10% from the previous year, according to our analysis of USDA documents (USDA, 2020), but Tyson framed this as an emergency: “We have a responsibility to feed our country. It is as essential as healthcare. This is a challenge that should not be ignored. Our plants must remain operational so that we can supply food to our families in America.”

The next day, Donald Trump signed an Executive Order invoking the Defense Production Act of 1950 to designate meatpacking facilities “critical infrastructure” and compel them to remain open. Meatpacking closures, he said, “threaten the continued functioning of the national meat and poultry supply chain, undermining critical infrastructure during the national emergency.” The Executive Order, which allowed the Department of Agriculture to invoke the Defense Production Act, did not explicitly mandate that plants stay open, but it signaled that the decisions around whether to close or reopen a plant should be driven by the federal government, not state authorities. It is worth noting here that in the context of the coronavirus pandemic response, the Trump Administration knowingly and intentionally made state governors compete with each other for ventilators, personal protective equipment (PPE), and other supplies under the pretext of empowering individual states (Gross, 2020). Nevertheless, when it came to the specific details of the meatpacking sector, Donald Trump acted on the advice of company CEOs to take decision-making power out of states’ hands after some governors and state health agency representatives recommended or required plant closures.

As a rationale for keeping plants open, the Executive Order (which did not mandate PPE for workers) cited the potential for nutritional deficiencies: “It is important that processors of beef, pork, and poultry in the food supply chain continue operating and fulfilling orders to ensure a continued supply of protein for Americans.” As workers are falling ill and dying, arguing that people must continue to have an adequate “supply of protein” is striking in a couple ways. The country, of course, has absolutely no shortage of protein—in fact, experts have argued for decades that people in the US on average consume much more meat and protein than we actually require (Lappé, 1971). This is hardly a nation inflicted with kwashiorkor. The Executive Order did not mandate that US production of peanut butter, quinoa, oatmeal, lentils, or garbanzo beans be ramped up to fulfill the potential “protein supply” crisis. Further, on-site managers at grocery stores and even analysts at the USDA reported that there were no significant shortages of pork in the midst of packing plant closures (Food & Water Watch, 2020). On the issue of Americans, specifically, needing meat, the President did not mention in his Executive Order that pork companies wanted their packing plants to remain open in large part to maintain current pork export levels, which account for one-third of total US production (Wilkie, Hirsch, & Repko, 2020). Even more telling about the rationale for the Executive Order, though, is that workers and their health are not even an afterthought in it. The President used this Order to ensure continued sales of meat and to ensure federal control over meatpacking closure decisions, but not to ensure or even address workers’ safety. Taken together, these actions suggest that a shortage of healthy workers is less important than a shortage of profitable exports. After May 1, in the face of the Executive Order the number of meatpacking closures quickly declined even while rates of infection continued to increase. The packing companies made use of the opportunity created by crisis to buy low (from farmers) and sell high (to grocery stores and restaurants), and the sale of meat in grocery stores grew during the pandemic more than sales of any other sector of product (Fassler & Brown, 2020). By July 10, 2020, 162 meatpacking workers had died.

### Criminogenic Capitalist Law and the Absence of Legal Liability

“We’re working with Tyson.” That is what the President of the United States said to a reporter on April 28 regarding the closure of meatpacking plants. He said he wanted to “solve any liability problems, where they had certain liability problems, and we’ll be in very good shape. We’re working with Tyson, which is one of the big companies in that world” (Wilkie, Hirsch, & Repko 2020). The next day he hosted a private phone call with meat company CEOs, including Tyson, Smithfield, and the National Pork Board (Schroeder, 2020). Trump had hosted a conference call on March 15 with some of the same companies. Reportedly, liability and export markets were among these companies’ major concerns.

Functionally, Donald Trump’s Executive Order federalizes the risk for meatpacking corporations to keep facilities open, transferring legal liability from the employers or from states to the US government. His order would presumably indemnify the companies if lawsuits were brought on behalf of workers. US Senator Majority Leader Mitch McConnell sought to further bolster this objective through legislative intervention (which is separate from the Executive Order), and in April 2020 asserted that there was an “urgent need” for Congress to pass federal legislation that would shield meatpackers and other businesses from legal liability. Republicans introduced the Protecting Protein Production and Consumers Access Act, which would specifically offer federal protection for meatpackers. The General Counsel for the National Association of Manufacturers, which lobbied Congress to make these corporate legal protections part of any subsequent economic stimulus bills, felt optimistic about approval: “There’s a real chance we can get these protections for our members” (Tankersley & Savage, 2020). *The Hill* reported that, “The business community has pressured the White House and Congress to include these protections in another round of coronavirus relief aid” (Gangitano, 2020), which was expected to be passed that fall but languished in large part because McConnell would not support aid for individuals and families struggling under the pandemic without legal assurances for corporations. “As Congress gears up for the next installment of its stimulus package,” *The New York Times* editorial board wrote on May 15, 2020, “Mr. McConnell has drawn a line: No more money for anyone until businesses get immunity from liability during the pandemic” (Board, 2020). Federal liability protections for private sector employers remained a divisive and contentious topic up through the signing of the last Trump administration COVID relief bill, finally passed by Congress on December 21, 2020 (Gregg & MacMillan, 2020; Martin, 2020; Mierzwinski, 2020; Riffs et al., 2020).

Corporate strategies to advocate for liability protections also went beyond congresspeople. Smithfield targeted its liability lobbying efforts on a network of Republican attorneys general across US states in 2020, and the company’s efforts appear to have been successful (Fang, 2020). Three days after receiving a $25,000 “donation” from Smithfield, the association of Republican attorneys general “sent a letter to Congress demanding broad business immunity from civil claims holding companies responsible for coronavirus infections due to working conditions.” Though federal assurances did not materialize, state legislatures took action. Iowa and North Carolina—two states with high concentration of pork packing facilities—passed “partial liability limit” waivers by July 2020, providing “sweeping immunity” to pork packing companies against COVID-related lawsuits.

## Analysis and Discussion

Industrial food systems are capital accumulation projects. That is their primary function, as agricultural regimes in the Global North have been shaped by the Euro-centric project of creating “elaborate commodity chains to feed themselves on the agricultural wealth of the entire world” (Ochoa, 2012, p. 24). It is a secondary feature that these industrial commodification regimes just so happen to actually feed some people—though surely not all or in equitable fashion (Escudero, 1991). The magnitude of what might properly fall within the purview of state-corporate harms within the food sector is virtually impossible to tackle in one analysis. In this paper we focused on actions and inactions of the state in bolstering corporate interests at the expense of laborers within the US pork industry.

The 15 days between April 13, when OSHA gave the greenlight to meatpacking companies to do their own investigations of the working conditions in their plants, and April 28, when the President of the United States signed an Executive Order compelling meatpacking plants to re-open and remain open, are highly significant. In between those dates, the President met with the most powerful corporate officers and lobbyists in the pork packing industry. In an Executive Order that was drafted by those lobbyists and meatpacking heads (Brabell & Yeung, 2020), he used the power of the federal government (both the executive branch and the USDA) to invoke law to coerce workers to remain at work in demonstrably unsafe workplaces.^9^ The US Chamber of Commerce—a “voice of business”—mocked protections that might keep workers safe and attempted to deflect attention away from workers’ illnesses and deaths to focus on a caricaturized version of a nanny state that capriciously doles out fines. Individual states and the US Congress acted in a manner that ensured the heightened precarity of workers, who, as a result of this state-corporate cooperation, were more likely to experience illness and death solely because a more robust and humane series of protections might disrupt the profit-maximizing activities of their employers.

In the actions taken by the pork packing industry and the state during the coronavirus pandemic, necropolitical power is on full display. Jose Andrade-Garcia, who was soon to retire but instead died because he had to go to work in a confined space where the virus was known to be in circulation, was drafted to die. No manager or state agent pointed at him and said, “It will be you,” but they chose him, nonetheless. He was chosen decades ago when OSHA determined that it would not regulate or even monitor the industrial use of an acid that irritates workers’ respiratory systems. He was chosen when OSHA decided it was okay for companies to investigate their own levels of safety, and when the safety manager in his plant determined that complaints about working conditions “lacked merit.” He was chosen when the President of the United States weaponized the law to insist that Andrade-Garcia’s plant remain open so that US exports would not decline, allowing his employer to coerce him to report to work. He was chosen by US Congresspeople, state legislators, governors, and even state attorneys general who, by arguing that liability legislation was necessary, implicitly acknowledged the reality that workers in pork packing plants were falling ill and dying, and that no pro bono, workers’ rights, immigrants’ rights, or human rights organization should successfully make the legal argument that his death was knowingly, willfully, and recklessly precipitated by the actions of powerful white men with deep pockets and connections. In each of these ways, the state-corporate enterprise has determined that souls such as Jose Andrade-Garcia’s can legally be subjected to the violence and death that accompany the accumulation of capital.

Workers are not “chosen” at random, and the composition of the workforce is critical to understanding how necropolitical power is organized in this setting. As noted, the majority of the front-line labor force in the industry are people of color. They are residents of communities in the South and Midwest that have seen meatpacking plants and factory farms open, introduce air and water pollution, and dominate wage setting (Ackerman & Musil, 2018). They are refugees who have come to the US from Vietnam, Haiti, Liberia, Mexico, Laos, Cuba, Sudan, Guatemala, and other countries where US policy and multinational corporate actions have contributed to the difficulty people have sustaining their families economically and keeping them safe (Chomsky, 2003; Chomsky & Herman, 1979; Galeano, 1971). They are immigrants who have been “secured” by pork packing companies’ “recruitment personnel,” otherwise known as coyotes (Stanley, 1992), and who are subjected to raids by Immigration and Customs Enforcement on a selective and instrumental basis. The plight of meatpacking workers is, empirically speaking, just one of many collateral consequences of border regimes and migration controls that are, themselves, reflective of state-corporate harm (Barak, 2021). In other words, border controls are not merely about policing the boundaries of citizenship and the demographic roster of the body politic. Migration controls are also about securing laborers. Foreign-born workers in the United States are simultaneously “essential” but marginalized, with approximately 74% of undocumented workers holding “essential worker” designations, and more undocumented workers than native-born in “essential worker” occupations (Kerwin & Warren, 2020, p. 282).

This simultaneous valorization-marginalization of migrant laborers is hardly unprecedented. In virtually every modern disease epidemic, the worst effects have been experienced by marginalized populations (Kapiriri & Ross, 2020), and the governmental responses are inherently political and politicizing. In the 2020 CARES Act provisions, for instance, the value of migrant labor is affirmed, but the value of migrant personhood denigrated and denied.^10^ With or without pandemics, the most exploitative jobs are generally held by individuals who are literally defined as non-agents in the political structure that governs their everyday lives. Frontline meatpacking is primarily a non-white occupational sector. With people of color and non-US citizens engaged in the most exploitative jobs, and these jobs quite literally being defined by the President as critical during a pandemic, it is evident that the state not only has an active stake COVID’s racially disparate outcomes, but an active role in making such outcomes an acceptable sacrifice in order to “get things back to normal.”

When we analyze whether the necropolitical racialization of labor is not only managed, but required, by the state, we start by asking if the state is engaging in performative legitimacy while simultaneously empowering the logics of capital. The racialization of the workforce refers to the deliberate creation of “undesirable exogenous others” who can be more easily contained, erased, terrorized, and removed (Glenn, 2015). When capital kills in racialized ways in which the state is invested, we witness the necropolitical racialization of labor.

In terms of liability—the question of who is responsible for workers’ deaths—it must be noted that while the corporate form enjoys many of the same rights and liberties as individual human beings (Teachout, 2014), corporations do not deal with the same criminal liability that individuals face, especially for offenses like homicide and manslaughter (Harlow, 2011; Ramirez & Ramirez, 2017). The practical consequences and significance of this socio-legal power asymmetry has been analyzed in US food systems, representing what León and Ken (2019, p. 38) call “criminogenic capitalist law,” or the legal, political, and ideological superstructure that enables food and agriculture industry giants to perpetuate various scales of fraud, harm, and ecological degradation. Criminogenic capitalist law is a conceptual framework for understanding how the laws, legal institutions, and various levers of governmental power are instrumentally shaped for the benefit of private sector interests, and in a manner antithetical to the public interest or the public commons (Chambliss & Seidman, 1971). When the US Senate Majority Leader and state attorneys general work to shield corporations from liability for the death of workers in pork packing plants—plants that are truly not “essential” or “critical” in any way—criminogenic capitalist law combines with the necropolitical racialization of labor to harrowing effect.

When private profits are pitted against human life, various levers of state power have aligned with protecting and responding to the former. In the necropolitical management of the economy, workers who are racialized and socio-legally defined as interchangeable can be subject to the most precarious of conditions in the name of capital interests. From this analytic perspective, COVID-19—within the boundaries of our case study—might be understood as a necropolitical magnifier, rendering state-corporate influence over life itself as more acutely visible.

## Conclusion

Rather than frame COVID-19 as a driver of the harms experienced by workers in the US pork packing sector, this case study reveals the current pandemic as a necropolitical magnifier, highlighting the criminogenic nature of our industrialized food systems. The actions of the state in 2020 demonstrate the symbiosis that allows workers in the pork packing industry to be confined and coerced to such an extent that they are rendered disposable, while meatpacking companies are legally defined and protected as indispensable from as high up as the executive branch.

There are compelling reasons to extend our analysis beyond the confines of the United States, and beyond the pork packing sector, but the present analysis is anchored to this specific context. As the actions and goals of a new presidential administration emerge in 2021, it may be tempting to view the events described in this paper as one-off outliers specific to an unconventional event and an unconventional presidential administration. Upon taking office, President Biden immediately undid many of the previous president’s executive orders and acted swiftly with Democrats in Congress to remediate some of the blunders of the previous year, enacting a vast COVID-19 relief bill in March 2021. What the state was able to do under Trump and McConnell may seem wholly different from what Democrats might have done in the same situation or will do moving forward. In terms of specific tactics and actions, this is likely accurate, and these differences do matter.

The state, though, does not eschew its ongoing capital accumulation project based on which party is in power. President Biden has not, as of this writing, repealed Trump’s Executive Order on meatpacking plants as critical infrastructure. He asked OSHA to issue “new guidance” to employers—not new rules, enforceable by law. Biden may or may not invite meatpacking company CEOs to meet with him and design legislation going forward, as Trump did, but he has accepted campaign contributions from these companies and individuals, as far as they can be tracked given current publicly available campaign finance data. Even a Democratic president will want meat exports to remain robust, meatpacking companies to stay in business, and profit in the industry to rise as part of a “healthy economy.” Such calculations and priorities are necessarily necropolitical, in that they cannot be accomplished without maintaining the legal infrastructure of capital that depends on disposable, replaceable, politically non-essential yet profitability-essential workers. As Biden’s priorities on immigration come into view, it is doubtful that any legislative initiatives from his administration will prevent or disincentivize employers in the pork packing industry from continually swapping out one set of vulnerable immigrant employees for another. These forms of slow, structural, legal violence against human beings require the ongoing and largely politically indifferent symbiosis between the capitalist state and its behemoth, profit-generating industries. For these reasons—magnified during a public health pandemic but also apparent in more “normal” times—the state’s role in the routinized corporate violence of confining workers such as Jose Andrade-Garcia within lethal working conditions and causing their deaths must not escape notice. If criminological inquiry remains tethered to measuring and pathologizing those who are most subject to formal capture and detection in systems of police, courts, and corrections, we will only delay our understanding of the vast scale of harms committed every day in service of state-corporate power.

## Notes

1. JBS and OSHA refused to reveal how many workers at the plant had been tested for the virus (Jett, 2020). The company did, however, have workers stand in line for a free meal as thanks for coming to work under dangerous conditions.

2. For scale, the Great Pyramid of Giza weighs 12 billion pounds.

3. Average weight of women and men, by proportion of meatpacking workforce (CDC, 2019).

4. There are 217,000 “frontline workers,” based on the precedent of categorization set by the Center for Economic and Policy Research (Fremstad et al., 2020). They include three occupations within the Animal Slaughtering and Processing Industry: Butchers and Other Meat, Poultry, and Fish Processing Workers (51-3020), Packaging and Filling Machine Operators and Tenders (51-9111), and Food Processing Workers, All Other (51-3099). We used these categories of US Bureau of Labor Statistics data (OES and CPS) for May 2019.

5. In the context of immigration law Menjívar and Abrego (2012, p. 1380) use the term *legal violence* “to capture the normalized but cumulative injurious effects of the law.” This perspective, along with prior literature on the criminogenic features of ostensibly legitimate food systems (see León & Ken, 2019), inform the present analysis of the meatpacking sector.

6. The US Department of Agriculture (USDA) Food Safety Inspection Service oversees safety in processing, storage, labeling, export, and transportation. The US Food and Drug Administration regulates the origin and distribution of food, and state departments of health regulate some food processing firms. Labor standards and working conditions in pork packing plants are supposed to be monitored by the federal and state offices of the Occupational Safety and Health Administration, and the National Labor Relations Board regulates union activity. As they pertain to pork packing workers, immigration laws are selectively enforced by US Immigration and Customs Enforcement under the US Department of Homeland Security. Finally, compliance with the Packers and Stockyards Act, which regulates the business practices—including antitrust activity—of meatpacking companies is monitored by the USDA Agricultural Marketing Service.

7. This state-centric bias extends to how crimes and harms of powerful actors are studied and understood within the social sciences generally and criminology specifically (see León, 2021; Michalowski, 2016).

8. An executive order is “a signed, written, and published directive from the President of the United States that manages operations of the federal government.” Executive orders have the force of law, although they do not require congressional approval and congress cannot overturn them (American Bar Association, 2021).

9. It might seem so ridiculous as to go without saying, but the President did not seek input from workers or their union prior to issuing the executive order (Brabell & Yeung, 2020).

10. Under the CARES Act, “a total of 6.2 million essential workers are not eligible for relief payments” (Kerwin & Warren, 2020, p. 282).
